# Serum Liver Fatty Acid Binding Protein Levels Correlate Positively with Obesity and Insulin Resistance in Chinese Young Adults

**DOI:** 10.1371/journal.pone.0048777

**Published:** 2012-11-07

**Authors:** Juan Shi, Yifei Zhang, Weiqiong Gu, Bin Cui, Min Xu, Qun Yan, Weiqing Wang, Guang Ning, Jie Hong

**Affiliations:** 1 Shanghai Clinical Center for Endocrine and Metabolic Diseases, Shanghai Institute of Endocrinology and Metabolism, Endocrine and Metabolic E-Institutes of Shanghai Universities (EISU) and Key Laboratory for Endocrinology and Metabolism of Chinese Health Ministry, Rui-jin Hospital, Shanghai Jiao-Tong University School of Medicine, Shanghai, China; 2 Laboratory of Endocrinology and Metabolism, Institute of Health Sciences, Shanghai Institutes for Biological Sciences, Chinese Academy of Sciences/Shanghai Jiao-Tong University School of Medicine, Shanghai, China; Warren Alpert Medical School of Brown University, United States of America

## Abstract

**Background:**

Liver fatty acid–binding protein (FABP1) plays an inconclusive role in adiposity. We investigated the association of serum FABP1 levels with obesity and insulin resistance in Chinese young people under 30 years old.

**Methodology and Principal Findings:**

Cross-sectional analysis including 200 obese and 172 normal-weight subjects matched for age and sex, anthropometric measurements were performed and serum FABP1 and biochemical characteristics were measured. Insulin resistance was determined by homeostasis model assessment of insulin resistance (HOMA-IR) and by the insulin sensitivity index (S_i_) derived from Bergman’s minimal model. FABP1 levels in obese subjects were significantly higher than those in normal-weight subjects (p<0.001) and the significance remained after adjustment for age, gender, alanine and aspartate aminotransferases (p<0.001). Serum FABP1 levels were significantly correlated with many metabolic-related parameters, with BMI and triglycerides as the independent determinants. FABP1 levels remained an independent risk factor of insulin resistance assessed by binary S_i_ (OR = 1.868 per SD unit, 95% CI [1.035–3.373], p* = *0.038) after adjustment for age, sex, BMI, waist circumference, systolic blood pressure, serum triacylglycerol, total cholesterol, HDL- and LDL-cholesterol,. FABP1 levels were also elevated with an increasing number of components of the metabolic syndrome (p for trend <0.001). Multiple regression modeling for the MetS and its components demonstrated that hypertriglyceridemia and low HDL-cholesterol were significantly correlated to serum FABP1 levels.

**Conclusions and Significance:**

Serum FABP1 correlates positively with obesity and insulin resistance in Chinese young adults. Our data supports the fact that FABP1 might be an important mediator participating in fatty acid metabolism and energy balance.

## Introduction

The fatty-acid-binding proteins (FABPs) are a family of low-molecular-weight intracellular lipid-binding proteins involved in the regulation of lipid metabolism and inflammation [1]. Cytosolic FABPs provide solubility and intracellular trafficking of long-chain fatty acids and other hydrophobic ligands [2], which are most active in long chain fatty acid (LCFA) uptake and metabolism (liver, intestine), oxidation (kidney, heart, skeletal muscle) and storage (adipose) [3], [4]. Since the initial discovery of FABPs in 1972, at least nine members have been identified. The family contains liver (L-), intestinal (I-), heart (H-), adipocyte (A-), epidermal (E-), ileal (Il-), brain (B-), myelin (M-) and testis (T-) FABPs [5]. In hepatocytes, adipocytes and cardiac myocytes, where fatty acids are prominent substrates for lipid biosynthesis, storage or breakdown, the respective FABPs make up between 1% and 5% of all soluble cytosolic proteins [6]. A growing body of evidence suggests that A-FABP circulates in human bloodstream and correlated closely with obesity and type 2 diabetes [7]. As another lipid chaperon of FABP family, L-FABP (called FABP1 hereafter) may also serve as an etiological mediator of obesity-related metabolic diseases.

FABP1 is abundant in the liver cytoplasm, but is also expressed in several other sites, including the intestine, pancreas, kidney, lung and stomach [8].Unlike the other members of the FABP family, FABP1 is able to bind two ligands simultaneously via two different binding sites with high and low affinities [9]. In addition, FABP1 can carry acyl-coenzyme A, eicosanoids, lysophospholipids, carcinogens, anticoagulants, such as warfarin, and haem, making it probably the most versatile chaperone in terms of its ligand repertoire [2]. Clear evidence on the specific impact of FABPs on cell biology and lipid metabolism in complex systems had been lacking until *FABP*-deficient mice models were created. Surprisingly, no change in appearance, gross morphology or viability was observed in *FABP1*-deficient mice [10], [11]. However, metabolic parameters in mice upon exposure to high-fat/cholesterol diet differed between studies [12]–[14]. Therefore, little consensus has been reached on the potential role of FABP1 in energy metabolism and obesity. Fatty acid binding proteins are released upon enterocyte membrane integrity loss. They are readily released into the circulation and renally cleared, which makes them useful as plasma and urine markers for enterocyte damage [15]–[18]. A study demonstrated that plasma FABP1 levels were detectable and could improve early diagnosis of intestinal ischemia [19]. In this study, we investigated the relationship of serum FABP1 levels with parameters of adiposity and insulin resistance in Chinese young adults.

## Methods

### Ethics Statement

This study was approved by the Institutional Review Board of the Ruijin Hospital, Shanghai Jiao Tong University School of Medicine and was in accordance with the principle of the Helsinki Declaration II. The written informed consent was obtained from each participant.

### Study Population

200 obese and 172 normal-weight subjects without significant health problems were included in our study. Included subjects were men and women between the ages of 17–30. All obese (BMI ≥30 kg/m^2^) subjects were collected consecutively (Jan 2008 to May 2010) from the specialized outpatient clinic for obesity in Ruijin Hospital, Shanghai JiaoTong University School of Medicine. Exclusion criteria for obese subjects included known diabetes, obesity due to known secondary causes, administration of endocrine hormones (including oral contraceptive pills and glucocorticoids), anti-hyperglycemic medications or lipid lowering drugs.

Conforming to the age and sex distribution in obese subjects, unrelated normal-weight subjects (n = 172) were all recruited from volunteers of Shanghai Jiao-Tong University School of Medicine on the basis of their normal BMI (<23 kg/m^2^) [20]. Exclusion criteria for normal-weight subjects included hypertension, impaired glucose regulation and hyperlipidemia.

### Clinical and Biochemical Measurements

All subjects underwent a standard 75-g OGTT at their first visit after an overnight fasting for at least 10–12 h between 07.00 and 08.00 hours in the morning. On the day of oral glucose tolerance test (OGTT), subjects were weighed in light clothing without shoes. Height and weight were measured by a height-weight scale, and body mass index (BMI, in kilograms per square meter) was calculated. Waist circumference (WC) was measured at the midpoint between the lower border of the rib cage and the top of the lateral border of the iliac crest. Blood pressure was measured at the right arm by a standard brachial cuff technique three times consecutively with 1 min intervals after at least 5 min rest in the seated position; the three readings were averaged for analysis.

On the day of the second visit (7 days apart from the day of OGTT), 129 obese and 22 normal-weight subjects participated in the frequently-sampled intravenous glucose tolerance test (FSIVGTT), which was performed according to those described in details elsewhere [21]. The insulin sensitivity index (S_i_) was calculated from the insulin-modified FSIVGTT using the Bergman’s minimal model equations. The repeatability and validity of S_i_ from FSIVGTT have been reported previously [22].

Glucose was measured immediately using an enzymatic method (Beckman CX-7 Biochemical Autoanalyser, Brea, CA, USA). Serum insulin was measured using a double antibody radioimmunoassay (DSL, Webster, Texas, USA). Serum total cholesterol and triglycerides were measured by enzymatic methods (Beckman coulter Inc, Fullerton, CA, USA). High-density lipoprotein-cholesterol (HDL-c) and low-density lipoprotein-cholesterol (LDL-c) were determined by immunoinhibition methods (HDL-c, LDL-c Direct, Wake Pure Chemical Industries Ltd. GmbH, Neuss, Germany). Serum alanine aminotransferase (ALT) and aspartate aminotransferase (AST) were measured using an automated Beckman Synchron clinical system CX5 PRO (Beckman Coulter, Brea, CA, USA) using Beckman Diagnostic reagents.

Overnight fasting serum samples were collected in tubes and stored at −80°C until serum FABP1 concentration assayment by human FABP1 ELISA kits developed in house using antibodies from R&D Research and Diagnostic Products, USA. Briefly, 100 µl sera and calibrators (human FABP1, SRP4501, Sigma-Aldrich Shanghai Trading Co., Ltd Century Ba-Shi Building 22A–B, 398 Huai Hai Zhong Road) were added to 96-well microplates coated with an anti-FABP1 antibody (MAB29641, R&D Systems, Minneapolis, MN, USA). Detection antibody (BAF2964, R&D Systems, Minneapolis, MN, USA) was biotinylated. A calibration curve was constructed by plotting the absorbance values at 492 nm vs. the FABP1 concentrations of the calibrators, and concentrations of serum samples were determined by using this calibration curve (Fig. S1). The intra- and inter-assay coefficients of variations (CVs) were <10% and <15%, respectively. The lower detection limit was 39.06 pg/ml.

### Definitions

Insulin resistance index (homeostasis model assessment of insulin resistance, HOMA-IR) was calculated using homeostasis model assessment methods, as fasting insulin (IU/ml) × fasting glucose (mmol/L)/22.5. Insulin resistance was defined as S_i_ <0.990.

The MetS was defined according to the U.S. National Cholesterol Education Program Adult Treatment Panel III (NCEP ATP III) guidelines [23] and modified as recommended in the latest American Heart Association/National Heart, Lung and Blood Institute Scientific Statement [24] by adopting the Asian criteria for waist circumference and a lower cutoff for fasting glucose. The MetS was defined as having three or more of the following metabolic risk factors: 1) central obesity (waist circumference ≥80 cm in females and ≥90 cm in males); 2) hypertriglyceridemia (fasting triglyceride ≥1.69 mmol/liter); 3) low HDL-cholesterol (fasting HDL<1.29 mmol/liter in females and <1.04 mmol/liter in males); 4) hyperglycemia (fasting glucose ≥5.6 mmol/liter or already on oral hypoglycemic agents for treatment of type 2 DM); and 5) hypertension (sitting blood pressure ≥130/85 mm Hg, taken as a mean of three readings obtained after resting for at least 5 min, or on regular antihypertensive medications).

### Statistical Analysis

Data were tested for normal distribution and logarithmically transformed for statistical analyses when required. Student *t* (for data that were continuous variables) or χ^2^ test (for data that was categorical variables) were used to compare between normal-weight and obese subjects. In addition, FABP1 levels in the two groups were further adjusted for comparison by univariate analysis of general linear model. We further adjusted for ALT and AST values in addition to age and gender considering the fact that FABP1 is abundant in liver cytoplasm and abnormal liver function may influence serum FABP1 levels. Correlations between FABP1 levels and other metabolic parameters were calculated by Spearman correlations coefficients. Multiple stepwise regression was applied to examine the independent predictors for FABP1 and HOMA-IR. Binary logistic regression was used to predict the risk factors for insulin resistance, the MetS and its components. When we applied multiple stepwise regression models to examine the association of FABP1 to insulin resistance, we considered the impact of demographic (age and gender), anthropometric (BMI, waist circumference, systolic blood pressure) and biochemical (lipids) parameters on insulin resistance. When we explored the correlation between FABP1 and the MetS and its components, we adjusted for age, gender and BMI. All analyses were performed using SPSS (version 13.0; Chicago, IL). For all tests, p values less than 0.05 were considered statistically significant.

## Results

### Baseline Characteristics of Subjects by Weight-Category

There were no significant differences in age and gender between obese and normal-weight subjects (Table 1). Compared with normal-weight subjects, obese subjects had higher BMI, WC, blood pressure, ALT, AST, TG, TC, LDL-c, fasting glucose and insulin, 2-hour glucose and HOMA-IR, as expected. Obese subjects also had lower levels of HDL-c and S_i_ (examined in 129 obese and 22 normal-weight subjects) than did normal-weight subjects (Table 1). LogFABP1 was significantly higher in obese subjects than in normal-weight subjects (2.76±0.12 pg/ml *vs*. 2.51±0.28 pg/ml; p<0.001). The significance remained even after adjusting confounding factors such as age, sex, ALT and AST values (p<0.001). There was no significant difference in FABP1 levels between genders.

**Table 1:** Baseline Demographics of Subjects by Weight Category.

	Normal-Weight	Obese	p-value
n	172	200	
Age (years)	23.3±2.6	22.8±3.4	0.057
Sex (n male/female)	61/111	71/129	0.994
Body mass index (kg/m^2^)	20.62±1.48	35.33±4.56	<0.001
Waist circumference (cm)	72.8±6.2	107.1±12.5	<0.001
Systolic blood pressure(mmHg)	109.6±10.4	123.8±13.4	<0.001
Diastolic blood pressure(mmHg)	70.0±8.2	81.3±11.5	<0.001
Alanine aminotransferase(IU/l)	15.0 (12.0–20.0)	41.0 (26.0–74.0)	<0.001
Aspartateaminotransferase (IU/l)	20.0 (17.0–23.0)	29.0 (22.0–41.0)	<0.001
Triglycerides (mmol/l)	0.72 (0.58–0.90)	1.70 (1.12–2.20)	<0.001
Total cholesterol (mmol/l)	4.08 (3.72–4.55)	4.49 (4.09–5.07)	<0.001
HDL-cholesterol (mmol/l)	1.59±0.26	1.14±0.23	<0.001
LDL-cholesterol (mmol/l)	2.30±0.54	2.88±0.67	<0.001
Fasting blood glucose(mmol/l)	4.7 (4.5–5.0)	5.1 (4.7–5.5)	<0.001
2-h blood glucose(mmol/l)	4.9 (4.5–5.6)	7.3 (5.9–8.4)	<0.001
Fasting insulin (µIU/L)	6.3 (4.3–8.3)	20.4 (14.7–26.9)	<0.001
HOMA-IR (µIU•mol/L^2^)	1.3 (0.9–1.7)	4.5 (3.2–6.5)	<0.001
S_i_ (×10^−4^ min^−1^/µU/ml)^a^	7.49 (4.78–10.47)	0.81 (0.41–1.40)	<0.001
LogFABP1 (pg/ml)	2.51±0.28	2.76±0.12	<0.001

Data are means ± SD or median (inter–quartile range).

a Superscript symbol represents data from 22 normal–weight and 129 obese subjects. p-values were calculated by student t or χ^2^ test.

### Correlates of Serum FABP1 Concentrations

**Figure 1:**
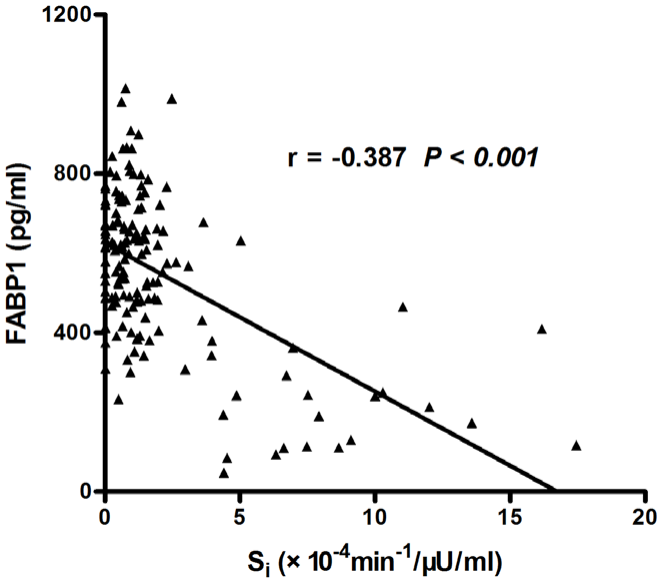
Correlation between insulin sensitivity index (S_i_) and FABP1 concentrations in a part of all the subjects (r = −0.310, *P*<0.001).

FABP1 levels correlated marginally with age (r = −0.104, p = 0.044). Noticeably, FABP1 was significantly associated with anthropomorphic measurements including BMI (r = 0.357, p<0.001), WC (r = 0.413, p<0.001), SBP (r = 0.242, p<0.001) and DBP (r = 0.169, p = 0.002), as well as lipid profile including TG (r = 0.422, p<0.001), TC (r = 0.137, p = 0.012), HDL-c (r = −0.424, p<0.001) and LDL-c (r = 0.191, p = 0.001). Among glucose homeostatic parameters, FABP1 was significantly associated with FBG (r = 0.126, p* = *0.016), fasting insulin (r = 0.471, p<0.001) and HOMA-IR (r = 0.461, p<0.001) (Table 2). Figure 1 shows the significant relationship between S_i_ and FABP1 in 129 obese and 22 normal-weight subjects (r = −0.387, p<0.0001). In stepwise regression analysis, BMI (β = 0.425, p<0.001) and TG (β = 0.183, p = 0.004) were independent determinants for serum logFABP1 concentrations after adjustment for all the covariables in Table 2.

**Table 2:** Spearman correlation and stepwise regression analysis in all the study subjects with FABP1 as a dependent variable.

Covariables	Spearman Correlation		Stepwise	
	r	p*-*value	β	p-value
Age (years)	−0.104	0.044	–	–
Sex	−0.056	0.284	–	–
Body mass index (kg/m^2^)	0.357	<0.001	0.425	<0.001
Waist circumference (cm)	0.413	<0.001	–	–
systolic blood pressure(mmHg)	0.242	<0.001	–	–
diastolic blood pressure (mmHg)	0.169	0.002	–	–
triglycerides (mmol/L)	0.422	<0.001	0.183	0.004
total cholesterol (mmol/L)	0.137	0.012	–	–
HDL-cholesterol (mmol/L)	−0.424	<0.001	–	–
LDL-cholesterol (mmol/L)	0.191	0.001	–	–
fasting blood glucose(mmol/L)	0.126	0.016	–	–
Fasting insulin (µIU/ml)	0.471	<0.001	–	–
HOMA-IR (µIU•mol/L^2^)	0.461	<0.001	–	–

r, Spearman correlation coefficient; β, Standardized regression coefficient; “−” indicates that the variable was not associated with FABP1 levels after adjustment for all the above variables in Table 2.

### Multiple Regression Modeling for Insulin Resistance

In univariate modeling, multiple parameters in addition to FABP1 were found to be significantly related to HOMA-IR, including BMI, WC, SBP, DBP, TG, TC, HDL-c, LDL-c, FBG, fasting insulin (all p<0.001, data not shown). FABP1 was not significantly associated (β = 0.072, p* = *0.111) with log HOMA-IR after controlling for age, sex, BMI, WC, SBP, serum triacylglycerol, total cholesterol, HDL- and LDL-cholesterol.

Insulin resistance was defined as S_i_ <0.990 (median of S_i_ in 129 obese and 22 normal-weight subjects). Binary logistic regression was performed to analyze the independent risk factors of insulin resistance. After adjusting age, sex, BMI, waist, SBP, serum triacylglycerol, total cholesterol, HDL- and LDL-cholesterol, FABP1 levels remained an independent risk factor of insulin resistance (OR = 1.868 per SD unit, 95% CI [1.035–3.373], p* = *0.038, Table 3).

**Table 3:** The Multiple Regression Modeling for binary insulin resistance and the MetS associated with per SD increase in serum FABP1.

Model	Adjustment	Binary Insulin Resistance		The MetS	
		OR (95% CI)	p-value	OR (95% CI)	p-value
Model 1	Adjusted for age and sex	2.279 (1.491–3.483)	<0.001	1.897 (1.452–2.480)	<0.001
Model 2	Further adjusted for BMI based on Model 1			1.242 (0.848–1.818)	0.265
Model 3	Further adjusted for WC and SBP based on Model 2	1.882 (1.123–3.157)	0.016		
Model 4	Further adjusted for serum triacylglycerol, total cholesterol, HDL- and LDL-cholesterol, based on Model 3	1.868 (1.035–3.373)	0.038		

Binary Insulin resistance was defined as S_i_ <0.990 (median of S_i_ in 129 obese and 22 normal-weight subjects). The MetS was defined according to the U.S. National Cholesterol Education Program Adult Treatment Panel III (NCEP ATP III) guidelines.

### Serum FABP1 and the MetS

**Figure 2:**
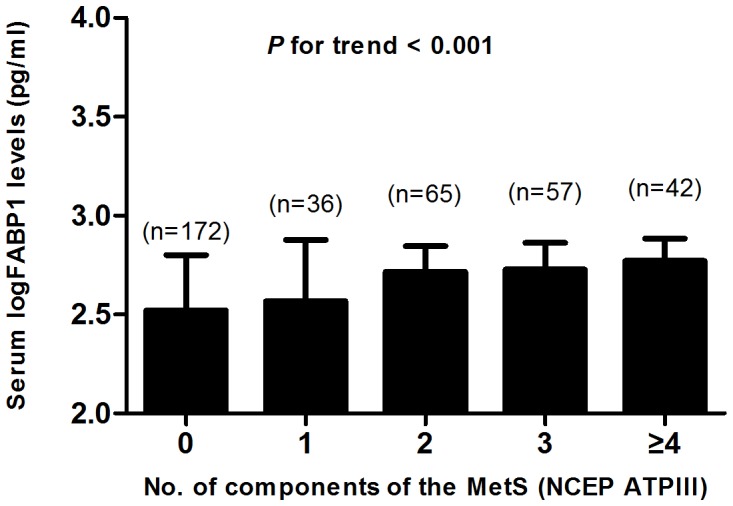
Serum concentrations of FABP1 elevated with increasing number of components of the MetS.

We examined the relationship between serum FABP1 levels and the MetS (NCEP ATPIII) in our study population. LogFABP1 was significantly higher in subjects with the MetS than in subjects without the MetS (2.75±0.12 pg/ml vs. 2.59±0.27 pg/ml; p<0.001). To further explore the relationship between FABP1 and the MetS, we stratified the mean levels of serum logFABP1 by the number of components of the MetS. This analysis showed that serum concentrations of logFABP1 elevated with increasing number of components of the MetS (p for trend <0.001) (Fig. 2). Multiple regression modeling for the MetS and its components (binary) demonstrated that hypertriglyceridemia (p = 0.007) and low HDL-cholesterol (p = 0.001) were significantly correlated to serum FABP1 levels (Table 3 and Table S1).

## Discussion

In this study, we show for the first time that serum FABP1 levels strongly relates to anthropomorphic measurements, lipid profile and glucose homeostatic parameters in young Chinese adults. Our multiple stepwise regression analysis has identified BMI and TG as significant independent contributors to serum logFABP1 levels. Moreover, the relationship between FABP1 and insulin resistance (reflected by binary S_i_) remains significant even after controlling for confounders. FABP1 levels were also elevated with an increasing number of components of the metabolic syndrome (p for trend <0.001). Multiple regression modeling for the MetS and its components demonstrated that hypertriglyceridemia and low HDL-cholesterol were significantly correlated to serum FABP1 levels. These novel findings suggest that serum FABP1 levels may act as a circulating biomarker of adiposity and insulin resistance-related metabolic diseases.

Our data demonstrate that FABP1 is markedly increased in healthy obese subjects versus normal-weight subjects, and that it correlates strongly with central adiposity. In line with our study, two *FABP1* ^−/−^ studies [13], [14] demonstrated that *FABP1* ^−/−^ mice are protected against obesity when fed a high fat diet. Because a review suggests that FABP1 could have an important role in preventing age- or diet-induced obesity [25], the “paradoxical” elevation of serum FABP1 in obese subjects might be a compensatory up-regulation of the human body to counteract the metabolic stress imposed by obesity. Alternatively, it is possible that obesity may cause resistance to FABP1 actions, leading to its compensatory up-regulation. Overall, given the cross-sectional nature of our study, no causal inference can be drawn.

There have been no reports on the association of serum FABP1 levels with insulin resistance in human. However, studies on FABP1 and lipid metabolism may provide clues as the relationship between these two parameters. Given that FABP1 is expressed at very high levels (2–5% of cytosolic protein) in hepatocytes [6] and that these levels correlate well with lipid metabolism [26], it can be speculated that FABP1 contributes considerably to hepatic lipid-binding and lipid metabolism. The association of FABP1 T94A polymorphism with fasting plasma triglycerides and LDL-c concentration in females support previous findings about the functional role of the FABP1 protein in fatty acid metabolism in the liver [27]. A recent study by Peng XE *et al*. [28] suggests that genetic variations within FABP1 influence susceptibility to non-alcohol fatty liver disease independently or jointly. Our study found TG was independently associated with serum FABP1 by multiple stepwise regression analysis. Additionally, hypertriglyceridemia and low HDL-cholesterol were significantly correlated to serum FABP1 levels after adjusting for age, gender and BMI. Therefore, it was proposed that ectopic deposition of TG in liver mediated by FABP1 may contribute to the insulin resistance in a certain respect. Our multiple regression modeling for insulin resistance suggested that lipids may mediate the relationship between FABP1 and insulin resistance. Further prospective studies are needed to test this speculation.

Another notable observation of the present study was the spearman correlation between FABP1 and fasting blood glucose in non-diabetic subjects. Clinical studies on FABP1 and glucose regulation are lacking. A study reported that the common Ala/Ala94 amino acid variant in FABP1 contributed significantly to decreased hepatic glycogenolysis and less severe hyperglycemia in lipid-challenged humans [29]. Urinary FABP1 accurately reflected the severity of diabetic nephropathy in type 2 diabetes [30]. Other findings suggest that increased FABP1 expression is associated with insulin-dependent diabetes and gestational diabetes in humans, streptozotocin-induced diabetes or obesity in rats, and type 1 diabetes in mice [31]–[34]. Further studies are needed to determine the role of FABP1 in modulating glucose metabolism.

Our study had some limitations. First, the source of serum FABP1 has not been established. Although it is abundant in the liver cytoplasm, but is also expressed in several other sites, including the intestine, pancreas, kidney, lung and stomach [8]. The specific function of FABP1 may be linked to its tissue distribution and intracellular localization. Second, the cross-sectional design of this study limits the interpretation of its results, especially with regard to cause-effect interactions. Third, this study was conducted in Chinese unrelated young people living in eastern area. However, because there may be differences in FABP1 expression based on genetic background, our conclusion needs to be drawn with additional studies on young people of different races.

In conclusion, we provided clinical evidence that serum FABP1 levels were positively associated with obesity and insulin resistance. However, phenotypic difference of *FABP1* ^−/−^ mice in metabolic parameters remains an unaddressed question of great importance. Thus, research exploring the role of FABP1 in adiposity combining clinical research and animal study is increasingly necessary.

## Supporting Information

Figure S1 A calibration curve was constructed by plotting the absorbance values at 492 nm vs. the FABP1 concentrations of the calibrators (y = 0.081+0.003*x−1.55E−06*x^2^+2.84E−10*x^3^, R^2^ = 0.999)
(DOC)

Table S1 The Multiple Regression Modeling for the individual components of the MetS associated with per SD increase in serum FABP1. The binary individual components of the MetS were defined according to the U.S. National Cholesterol Education Program Adult Treatment Panel III (NCEP ATP III) guidelines.
(DOC)
