# Supplementary material for: Serum Liver Fatty Acid Binding Protein Levels Correlate Positively with Obesity and Insulin Resistance in Chinese Young Adults
Source: PLoS One. 2012 Nov 7;7(11):e48777. doi: 10.1371/journal.pone.0048777 (PMC3492433; doi:10.1371/journal.pone.0048777)
Supplement: Figure S1 — A calibration curve was constructed by plotting the absorbance values at 492 nm vs. the FABP1 concentrations of the calibrators (y = 0.081+0.003*x−1.55E−06*x2+2.84E−10*x3, R2 = 0.999) (DOC) [file pone.0048777.s001.doc]

**Figure S1.** Calibration curve
