# Supplementary material for: Serum Liver Fatty Acid Binding Protein Levels Correlate Positively with Obesity and Insulin Resistance in Chinese Young Adults
Source: PLoS One. 2012 Nov 7;7(11):e48777. doi: 10.1371/journal.pone.0048777 (PMC3492433; doi:10.1371/journal.pone.0048777)
Supplement: Table S1 — The Multiple Regression Modeling for the individual components of the MetS associated with per SD increase in serum FABP1. The binary individual components of the MetS were defined according to the U.S. National Cholesterol Education Program Adult Treatment Panel III (NCEP ATP III) guidelines. (DOC) [file pone.0048777.s002.doc]

**Table S1.** The Multiple Regression Modeling for the individual components of the MetS associated with per SD increase in serum FABP1

| Model | Adjustment | Central obesity | | Hypertriglyceridemia | | Low HDL-cholesterol | | Hyperglycemia | | Hypertension | | |
| --- | --- | --- | --- | --- | --- | --- | --- | --- | --- | --- | --- | --- |
|  |  | OR (95% CI) | p-value | OR (95% CI) | p-value | OR (95% CI) | p-value | OR (95% CI) | p-value | OR (95% CI) | p-value | |
| Model 1 | Adjusted for age and sex | 3.890 (2.811-5.384) | < 0.001 | 2.103 (1.589-2.783) | < 0.001 | 2.552 (1.918-3.394) | < 0.001 | 1.686 (1.216-2.338) | 0.002 | 1.320 (1.016-1.716) | 0.038 | |
| Model 2 | Further adjusted for BMI based on Model 1 | 0.927 (0.177-4.863) | 0.928 | 1.627 (1.144-2.314) | 0.007 | 1.807 (1.278-2.554) | 0.001 | 1.327 (0.899-1.960) | 0.154 | 0.950 (0.691-1.307) | 0.754 |  |

The binary individual components of the MetS were defined according to the U.S. National Cholesterol Education Program Adult Treatment Panel III (NCEP ATP III) guidelines.
